# Utilization and Impact of a Radiation Nursing Clinic to Address Acute Care Needs for Patients with Gynecologic Cancers

**DOI:** 10.3390/curroncol31030125

**Published:** 2024-03-21

**Authors:** Aaron Dou, Genevieve Bouchard-Fortier, Kathy Han, Michael Milosevic, Jelena Lukovic, Stephanie L’heureux, Xuan Li, Mary C. Doherty, Jennifer Croke

**Affiliations:** 1 Radiation Medicine Program, Princess Margaret Hospital Cancer Centre, Toronto, ON M5G 2M9, Canada; 2 Department of Gynecologic Oncology, Princess Margaret Hospital Cancer Centre, Toronto, ON M5G 2M9, Canada; 3 Department of Radiation Oncology, University of Toronto, Toronto, ON M5S 1A1, Canada; 4 Department of Medical Oncology and Hematology, Princess Margaret Hospital Cancer Centre, Toronto, ON M5G 2M9, Canada; 5 Department of Biostatistics, Princess Margaret Hospital Cancer Centre, Toronto, ON M5G 2M9, Canada

**Keywords:** gynecologic cancers, radiotherapy, concurrent chemoradiation, acute care utilization, supportive care

## Abstract

Background: The risk factors for acute care utilization in gynecologic oncology patients are poorly understood. This study aimed to evaluate risk factors for the utilization of our centre’s acute care radiation nursing clinic (RNC) by gynecologic oncology patients receiving radiotherapy (RT). Methods: This was a retrospective cohort study of gynecological cancer patients treated with RT at an academic cancer centre between 1 August 2021 and 31 January 2022. Data on socio-demographics, clinical and treatment characteristics, and RNC visits were collected and summarized by descriptive statistics. The Wilcoxon rank sum test and chi-squared test/Fisher’s exact test were used for comparisons of continuous and categorical variables, respectively. Results: RT was delivered to 180 patients, of whom 42 (23%) received concurrent chemoradiation (CCR). Compared to those receiving RT alone, patients receiving CCR had higher rates of RNC utilization (55% vs. 19%, *p* < 0.001). Within the CCR cohort, patients who presented to the RNC were more likely to be unpartnered (43% vs. 11%, *p* = 0.04), receive a referral to Psychosocial Oncology (39% vs. 5.3%, *p* = 0.01), and experience treatment interruptions (52% vs. 16%, *p* = 0.02). There were no associations between RNC visits and age, disease site, or distance from the cancer centre. Conclusions: The receipt of CCR and specific psychosocial risk factors were associated with increased RNC utilization. Targeted strategies and early intervention to better meet the supportive care and psychosocial needs of this vulnerable population are needed.

## 1. Introduction

Randomized studies have shown that concurrent chemoradiotherapy (CCR) regimens improve cancer outcomes compared to radiotherapy alone for patients with gynecological malignancies, including locally advanced cervical cancer and high-risk endometrial cancers [1,2,3,4,5,6,7]. Despite these improvements, gynecologic cancer patients undergoing concurrent chemoradiotherapy experience a high symptom burden and increased adverse effects due to the synergistic toxicity of the two treatment modalities [8,9].

Treatment-related toxicities may be both acute and long-lasting, leading not only to physical symptoms but also to psychosocial distress and financial toxicity [10]. This, in turn, results in greater utilization of acute care resources, including emergency department (ED) visits and hospitalizations, increasing healthcare system costs [10]. To address the acute care needs of cancer patients undergoing radiotherapy, our institution has established a radiation nursing clinic (RNC). The RNC is a walk-in clinic—the first of its kind in Canada—that specializes in the management of acute toxicities during cancer treatment. The RNC is available to all patients receiving radiation therapy and serves the clinical, practical, educational, and psychosocial needs of patients in one setting, thereby allowing for increased continuity and streamlining of care. Patients attending the RNC usually receive multiple interventions. For example, a patient may receive intravenous hydration with anti-emetic pharmacotherapy, laboratory investigations for electrolyte derangements, and patient education on non-pharmacologic strategies for preventing dehydration. The RNC diverts patients who would otherwise have been treated in the emergency department or the radiation review clinic, neither of which can provide the entire suite of services available in the RNC. The former is not an ideal forum to address the psychosocial and educational needs of the patient or to provide multi-day follow-ups, and the latter is unable to provide close continuous monitoring of the patient and to implement higher-acuity treatments, such as intravenous medication or hydration.

Early symptom identification has been shown to improve survival, reduce unplanned acute care utilization rates, and decrease treatment interruptions in select cancer patient populations [11,12,13,14]. Within the head and neck cancer population, studies have shown that patients undergoing concurrent chemoradiotherapy experience a high symptom burden in addition to financial toxicity [15]. Increased age, baseline frailty, concurrent chemoradiotherapy, the presence of comorbidities, and low socioeconomic status have been identified as risk factors for acute care utilization, including emergency department visits and hospital admission [16,17,18,19,20,21]. These results are corroborated by studies in patients with glioblastoma, which also demonstrated an association between acute care utilization and age, Karnofsky Performance Status (KPS), and concurrent chemoradiotherapy [22,23].

Despite the high symptom burden that patients with gynecologic cancers experience, there is a paucity of literature exploring the clinical, demographic, and psychosocial risk factors for additional supportive care and acute care utilization. Indeed, there have been minimal recent studies identifying patients at risk for acute care utilization during treatment, which in turn affects treatment adherence, treatment interruptions, as well as long-term disease outcomes. Thus, the identification of risk factors for acute care utilization will permit earlier identification of the patients who are most vulnerable to treatment-related toxicity and complications and is of critical importance to the systematic development and improvement of targeted supportive care interventions for gynecologic oncology patients. Given the availability of the RNC at our centre as an early acute care resource for patients, we aimed to not only evaluate the risk factors for RNC utilization in a cohort of patients with gynecologic malignancies but also explore the downstream impact of the RNC on emergency departments’ visit frequency and hospitalizations.

## 2. Materials and Methods

### 2.1. Study Population

This was a single-centre retrospective cohort study as part of a quality improvement project at a tertiary cancer centre. Patients with biopsy-proven gynecological cancers (e.g., endometrial, cervical, vaginal, vulvar, ovarian) who were treated with radiotherapy between 1 August 2021 and 31 January 2022 were identified. Patients who did not complete their prescribed radiation course or experienced treatment interruptions were included in the analysis. This work was conducted as part of a quality improvement initiative and was exempt from our institutional research ethics board (QI ID#: 22-0408).

### 2.2. Study Setting

In the ambulatory oncology setting at the Princess Margaret Cancer Centre in Toronto, Canada, there is a nurse-led radiation oncology clinic, the Radiation Nursing Clinic (RNC). The RNC is open to patients from the start of their radiotherapy up until two weeks after their treatment is complete. The RNC provides a setting where patients can be assessed by a registered nurse and/or nurse practitioner for new or ongoing problems in the period between their weekly review appointments with the radiation oncologist. The RNC is the only clinic of its kind in Canada and is uniquely positioned to provide early supportive care to patients, including patient education, laboratory investigations, fluids, and pharmacologic treatment, with the goal of reducing downstream utilization of emergency department and inpatient resources.

### 2.3. Data Collection

Patient charts were retrospectively reviewed to collect data on (1) socio-demographics; (2) clinical characteristics (e.g., tumour site, FIGO stage, treatment intent); (3) treatment characteristics, including interruptions and/or adjustments; and (4) the number and timing of RNC visits. For patients who visited the RNC, information regarding the number of visits, chief complaint, length of stay, interventions performed, and disposition was also collected. Disposition outcomes from the RNC were defined as discharge home, transfer to an emergency department, or direct admission to an inpatient unit.

### 2.4. Data Analysis

Summary statistics were reported to describe socio-demographical and clinical characteristics by cohorts and for all patients. Wilcoxon rank sum test and chi-squared test/Fisher’s exact test were used for comparisons of continuous and categorical variables, respectively. A two-sided α of 0.05 was chosen as the threshold for statistical significance. All statistical analyses were performed using R version 4.2.2.

## 3. Results

### 3.1. Summary of Study Cohort

The baseline patient and treatment characteristics are presented in Table 1. Of the 180 patients eligible for inclusion, 42 (23%) received concurrent chemoradiation (CCR), and 138 (77%) received radiation therapy (RT) alone or as part of sequential chemoradiation/sandwich chemoradiation regimens.

**Figure 1:**
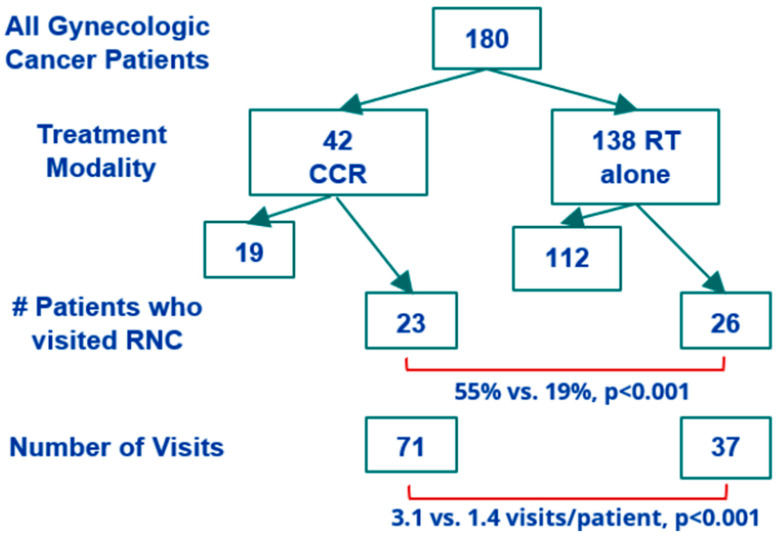
Summary of study design.

The two groups did not differ significantly in age. None of the patients in the CCR group had metastatic (Stage IV) disease, compared with 58 (42%) of the patients in the RT group. Thus, a greater proportion of patients in the RT group received radiation with palliative intent (47%) compared with zero patients in the CCR group. Comparatively, in the CCR group, the most common regimen was PORTEC-3 for endometrial cancer (62%). PORTEC-3 involves two phases of treatment: in the first phase, 5 weeks of external beam radiotherapy (4500 Gy in 25 fractions) are delivered, during which concurrent cisplatin is given to patients on weeks 1 and 4. Upon completion of radiation treatment, the second phase of PORTEC-3 involves adjuvant carboplatin-taxol chemotherapy. The second most common regimen after PORTEC-3 was curative intent external beam radiotherapy with weekly cisplatin for locally advanced cervical cancer (36%), also delivered as 4500 Gy in 25 fractions for most patients; three patients with cervix cancer in the CCR group (7%) received a simultaneous integrated boost (SIB) to a total dose of 5500 Gy in 25 fractions. The overall study design is summarized in Figure 1.

### 3.2. Risk Factors for RNC Utilization

Significantly more patients in the CCR group had at least one visit to the RNC compared to the RT group (55% vs. 19%, *p* < 0.001). CCR patients who visited the RNC were more likely to also be referred to Psychosocial Oncology (39% vs. 5.3%, *p* = 0.01). The Psychosocial Oncology Program at our institution is a multidisciplinary resource staffed by psychiatrists and allied health professionals that aims to specifically address the social, practical, psychological, emotional, spiritual, functional, and quality-of-life impact of cancer on patients and their families. Additionally, CCR group patients who visited the RNC were less likely to be partnered (57% vs. 89%, *p* = 0.04) and more likely to experience a treatment interruption (52% vs. 16%, *p* = 0.02) compared to CCR patients who did not visit the RNC. There was no association between age, disease site, disease stage, or distance from the cancer centre and RNC visits. Furthermore, the receipt of brachytherapy or radiation treatment to the para-aortic nodes was not associated with the rate of RNC utilization. A disease-free status at one-year post-completion of radiation treatment was also not associated with RNC utilization rates (Table 2).

### 3.3. RNC Visit Data

Of the 180 total patients in our cohort, 59 visited the RNC, for a total of 108 visits. CCR patients visited 71 times in total (3.1 visits per patient), and RT patients visited 37 times in total (1.4 visits per patient, *p* < 0.001). Patients with endometrial cancer receiving PORTEC-3 visited 43 times in total, of which 28 visits (65%) occurred during weeks 1 and 4 of treatment, corresponding to the weeks during which chemotherapy was delivered. Contrastingly, for cervical cancer patients receiving weekly cisplatin and radiation, 13 of 18 visits (73%) occurred during the final two weeks of treatment, with 10 visits (56%) occurring during the final week of treatment. All patients in the CCR group receiving PORTEC-3 for endometrial cancer and cisplatin + RT for cervical cancer received the same total dose of 4500 Gy over 25 fractions, with the exception of three patients with cervix cancer who received an SIB to a total dose of 5500 Gy in 25 fractions.

For patients in the CCR group, the majority of RNC visits were directed by radiation oncologists (following assessment in the radiation review clinic; 49%) and self-referrals by patients (42%). The remainder of the visits (9%) originated from radiation therapists (RTTs) at the treatment units. For patients in the RT group, 68% of visits were self-referrals, while those directed by radiation oncologists and RTTs each comprised 16% of the total visits. CCR patients most commonly presented to the RNC with symptoms of dehydration and received intravenous rehydration as the primary intervention (58% of visits), whereas RT-only patients presented most frequently with symptom management questions requiring patient education and without the need for additional treatment or investigations (41% of visits). The proportion of visits for which different interventions were performed is illustrated in Table 3 for both the CCR and RT groups. The majority of patients (96%) in both groups were discharged home from the RNC. The remaining 4% were either referred to the emergency department or admitted.

## 4. Discussion

In this study, we characterized the socio-demographic and clinical risk factors for RNC utilization in gynecologic cancer patients receiving RT. RNC utilization was significantly associated with the receipt of concurrent chemoradiation (CCR) regimens. Within the CCR cohort, unpartnered relationship status, treatment interruptions, and referral to the Psychosocial Oncology Program were correlated with increased RNC utilization.

Our data are consistent with previous work demonstrating the association between CCR regimens and increased acute care utilization in other cancer populations, specifically the head and neck oncology cancer population, which also experiences a high symptom burden from radiation treatment to sensitive structures [17,18,19,20]. This highlights not only the vulnerability of this target population but also the opportunity for healthcare resource optimization by proactively rather than reactively addressing patient needs. This will, in turn, reduce rates of downstream emergency department utilization and hospitalization as well as healthcare costs. For patients receiving CCR, we identified important psychosocial risk factors associated with RNC utilization, including unpartnered marital status and referral for psychosocial oncology assessment. Previous work has shown that caregivers play a crucial role in promoting self-care and supporting patient needs, not only for cancer patients but also for those with other chronic diseases; indeed, a lack of social support is a predictor of poor outcomes [24,25,26,27,28]. The concept of psychosocial vulnerability has not been previously identified as a risk factor for acute care utilization, specifically within oncology populations. Thus, it is important that providers aim to identify patients at increased risk for social isolation, as well as encourage caregiver engagement during the treatment process whenever possible. One strategy to do so is through utilizing patient-reported outcomes (PROs), which have been shown to facilitate oncologist–patient communication, decrease symptom burden, enhance supportive care measures, improve quality of life, and increase overall survival [29,30,31]. PROs may provide valuable information on the extent to which patients feel supported throughout their treatment. Additionally, the utilization of the Psychosocial Oncology Program illustrates the utility and necessity of integrated interdisciplinary care, in which the collaborative care of physicians from different disciplines, as well as allied health professionals, is needed to address the complex supportive care needs of cancer patients.

In our study, RNC utilization was not associated with age. This is in contrast to previous work that identified age as a predictor of symptom burden and acute care utilization in other cancer populations [32]. One potential explanation is that age may not be the most important factor for predicting symptom burden, but rather pretreatment frailty, a holistic measure that integrates both medical comorbidities and psycho-socioeconomic context [20]. Frailty has been variably defined in the literature: early definitions, such as that proposed by Fried et al. in 2001, focused upon the physical domain, defining the frailty syndrome using five criteria: (1) unintentional weight loss >10 lbs in the past year, (2) self-reported exhaustion, (3) weakness, indicated by weak grip strength, (4) slow walking speed, and (5) low physical activity [33]. Other work has expanded upon this definition to include psychological, cognitive, and social domains of frailty (e.g., the Rockwood Frailty Index); while frailty generally increases with age, age alone cannot predict an individual’s frailty [34]. The complex factors underlying frailty support the importance of addressing psychosocial vulnerability in cancer patients as a mechanism by which to reduce acute care utilization. Another explanation for the absence of correlation between age and acute care utilization in our study is that the average age of cervical cancer patients is lower than that of endometrial cancers; therefore, our sample size may be underpowered to identify associations between age and RNC utilization within disease sites.

Our data also showed no correlation between distance from the cancer centre and RNC utilization. Another study has shown that patients from rural backgrounds receiving CCR for cancer of the cervix experience greater acute toxicities [35]. Given that nearly all patients in our study lived within 100 km of the cancer centre, which is a large tertiary care centre, we may be under-representing this vulnerable population in our study; future work is recommended to identify their supportive care needs.

Specific clinical factors, including receipt of brachytherapy, radiation to the para-aortic nodes (which is associated with higher-stage disease), and disease-free status at one year following completion of radiation treatment, were not associated with RNC utilization. These findings must be interpreted with caution given the sample size limitations of this study as well as the heterogeneity of tumour anatomic sites, staging guidelines, and pathophysiology within gynecologic oncology. Nevertheless, the absence of an association between these clinical factors and acute care utilization does yield several important insights. In this study, only 180 gynecologic cancer patients were treated over a 6-month interval at a large tertiary cancer centre; as such, longer study times would be required to achieve sufficient power to stratify patients by tumour site/stages, treatment regimen, and other clinical factors. Given the aforementioned heterogeneity of gynecologic cancers, it may be more practical and impactful to first target the psychological and socioeconomic risk factors that are common to all patients with gynecologic cancer as a mechanism for identifying high-risk patients for acute care utilization. Additionally, both treatment guidelines and supportive care resources for oncology patients are constantly evolving, which may present challenges that confound the analysis of a more prolonged study. Thus, the psychosocial risk factors that this study identifies provide a preliminary area of intervention for addressing the supportive care needs of gynecologic oncology patients.

The majority of patients in both the CCR and RT groups presented to the RNC either through self-referral or were directed by their oncologist in the weekly radiation review clinic. Only a minority of patients (<10% for CCR patients) were directed by their RTT to the treatment unit. This represents a potential target area to reduce acute care utilization, given that RTTs interact with patients daily during radiation treatment and are thus uniquely positioned to proactively identify symptoms of concern. A novel, proof-of-concept study was recently performed at our centre, pairing complex breast cancer patients with one primary radiation therapist, known as a person-centred model of care. The authors found a 20% reduction in RNC visits during radiotherapy and a 50% reduction in patient hand-offs by radiation therapists [36]. A similar system could be trialed for high-risk gynecologic oncology patients undergoing concurrent chemoradiotherapy to improve continuity of care and provide daily symptom monitoring. Work is ongoing at our institution to explore the utility of this model of patient care in other cancer populations.

Our study also illustrated the impact of the RNC as an acute care resource for patients with gynecologic cancer. The RNC provides a setting for patients to efficiently receive multidisciplinary care for disease symptoms and treatment-related toxicity and is closely integrated with the patient’s primary radiation oncologist. This allows for greater coordination of care and more streamlined communication, with the goal of preventing escalation to the ED and/or hospitalization through proactive symptom assessment and management. Our study supported the utility of the RNC as a supportive acute care resource for patients, as only 4% of patients presenting to the RNC required an escalation of care to the ED or hospitalization. Without the RNC, a greater proportion of patients may have instead presented to the ED later in their treatment course with a greater symptom burden, not only affecting patient quality of life and disease outcome but also healthcare expenditures.

Our results support personalizing the timing and nature of supportive interventions to the specific needs and treatment regimen of each patient: for example, endometrial cancer patients receiving PORTEC-3 presented most frequently in weeks 1 and 4 of radiation treatment (65% of all visits), a few days after receiving chemotherapy. This is in contrast to cervical cancer patients receiving cisplatin + RT, who most often presented later in their treatment course (50% of visits during the final week of treatment), presumably due to the effect of cumulative toxicity. Thus, support for patients on PORTEC-3 may involve proactive hydration and nausea management immediately following chemotherapy, whereas support for cervical cancer patients may include more intense monitoring and symptom management as they progress through treatment.

The findings of this study need to be interpreted in the context of several important limitations. Firstly, as the study is observational, we cannot draw any conclusions of causation regarding the risk factors for unscheduled acute care visits. Additionally, this is a single-institution retrospective study covering a defined time period, limiting the ability to extrapolate findings to other cancer patients from different centres, especially those from rural settings. Given our focused patient population, the sample size limited our ability to stratify patients by disease site and stage when comparing risk factors and acute care utilization rates. As the fractionation schedule differs between different tumour sites and stages, this may affect the length of time during which patients can visit the RNC. As mentioned previously, future work may aim to recruit a larger sample size over a longer period of time to provide sufficient power to stratify patients by different clinical factors, with the caveat that such a study may be confounded by evolving clinical guidelines and changes in available supportive care resources. Lastly, we were unable to obtain adequate data regarding patient-reported outcomes, pretreatment frailty/comorbidities, ethnicity, education level, and primary level.

Nevertheless, the trends that we have highlighted through descriptive analysis—and, in particular, the importance of psychosocial risk factors—will assist clinicians and cancer centres alike in identifying vulnerable patients at risk for acute toxicity and acute care utilization during treatment. There has been a dearth of recent studies on acute care utilization in gynecologic cancer patients; the majority of literature published on risk factors for acute care utilization has been in the field of head and neck oncology due to the high symptom burden of these patients. Additionally, despite the similar supportive care needs and treatment-related toxicity that patients with gynecologic cancers experience, the heterogeneity of gynecologic malignancies increases the difficulty of studying this patient population. Recognition of the importance of psychosocial risk factors for acute care utilization is emerging, and recent literature has identified race and socioeconomic status as important predictors of acute care for gynecologic cancer patients [37]. Future work will expand upon our study and similar work to affirm the importance of these factors, as well as identify novel factors associated with acute care utilization.

## 5. Conclusions

In conclusion, this study identified several socio-demographic and clinical risk factors for RNC utilization in gynecologic oncology patients receiving RT and emphasized the value of the RNC in providing early symptom management for patients, especially those receiving CCR. Work is ongoing at our institution to implement a proactive remote symptom monitoring program for gynecologic oncology patients undergoing CCR. Future supportive care interventions should employ an interdisciplinary approach to identify and support high-risk cancer patients throughout the entire spectrum of their cancer journey to improve quality of life, patient outcomes, and healthcare resource utilization.

## Figures and Tables

**Table 1:** Patient and treatment characteristics for the study cohort.

		CCR Group (n = 42)	RT Group (n = 138)
Mean Age (SD)		59 (11)	65 (13)
Disease Site (%)	Cervix	15 (36)	28 (20)
	Uterus	26 (62)	94 (68)
	Vulva	1 (2)	3 (2)
	Ovary	0	13 (9)
Metastatic Disease (%)	Yes	0	58 (42)
	No	41 (98)	78 (57)
	Unknown	1 (2)	2 (1)
Treatment Intent (%)	Definitive	12 (29)	20 (14)
	Adjuvant	30 (71)	50 (36)
	Palliative	0	65 (47)
	Neoadjuvant	0	3 (2)
RNC Utilization (%)	Yes	23 (55)	26 (19)
	No	19 (45)	112 (81)

**Table 2:** Risk factors for acute care utilization in patients undergoing concurrent chemoradiotherapy (CCR).

	Visited RNC (n = 23)	Did Not Visit RNC (n = 19)	*p*-Value
Mean age (SD)	58 (11)	60 (11)	0.64
Mean distance from cancer centre (km) (SD)	34 (35)	21 (17)	0.26
Partnered marital status (%)	13 (57)	17 (89)	0.04 *
Treatment interruptions (%)	12 (52)	3 (16)	0.02 *
Psychosocial oncology referral (%)	9 (39)	1 (5.3)	0.01 *
Brachytherapy (%)	10 (43)	8 (42)	1.00
Para-aortic nodal radiation (%)	1 (4.4)	2 (11)	0.44
Disease-free at 1 year (%)	20 (87)	16 (84)	0.80

* Indicates statistical significance.

**Table 3:** Frequency of interventions performed in the RNC.

Intervention	CCR Cohort (n = 71 Visits)	RT Cohort (n = 37 Visits)
IV fluids	41 (58%)	9 (24%)
Pharmacologic	28 (39%)	8 (22%)
Laboratory investigations	23 (32%)	8 (22%)
Patient education only	4 (6%)	15 (41%)

## Data Availability

The data presented in this study are available on request from the corresponding author. The data are not publicly available to protect patient privacy.
